# A field-based general framework to simulate fluids in parallel and the framework’s application to a matrix acidization simulation

**DOI:** 10.1371/journal.pone.0261134

**Published:** 2022-02-03

**Authors:** Yuanqing Wu, Shuyu Sun

**Affiliations:** 1 College of Mathematics and Statistics, Shenzhen University, Shenzhen, Guangdong, China; 2 Computational Transport Phenomena Laboratory (CTPL), Division of Physical Sciences and Engineering (PSE), King Abdullah University of Science and Technology (KAUST), Thuwal, Saudi Arabia; Central State University & Ohio University, UNITED STATES

## Abstract

On the basis of numerous fluid simulation experiences, researchers have discovered that many common operations can be abstracted to form a general fluid simulation framework. These operations include the discretization of equations and variables, the computation of the coefficients, the assembly of the linear or nonlinear systems, the solving of the systems, etc. Furthermore, all of the operations can be attributed to the operations “in the field”, which is an abstract concept derived from the equations and variables. Thus, fluid simulations can be performed under a field-based general framework. Moreover, in response to the urgent need for large-scale fluid simulations, parallelism is integrated into the framework. Due to the convenience of the field operations, parallelization of the framework can be realized on both the OpenMP and MPI levels. In other words, because of the newly defined “fields”, a series of operations in fluid simulations can be simplified and unified. However, very few studies have noted this advantage, and therefore, this work attempts to fill the void. With the help of a field-based general framework, it is anticipated that the parallel codes of fluid simulations can be generated easily and quickly. As an application of the general framework, a parallel 3D simulator for matrix acidization called Masor is developed. The simulation results are regarded as physically reasonable by many studies, which verifies the correctness and effectiveness of the general framework. In addition, it is noteworthy that the parallel performance of Masor is decided by a solver.

## 1. Introduction

Computational fluid dynamics (CFD) [1, 2] focuses on the numerical simulation of fluids using computer technology. The general procedure undertaken for this kind of simulation is to first discretize the mathematical model with numerical schemes. After this step, if there are some implicit schemes used, a series of linear systems are formed. Although some works may generate nonlinear systems and use Newton’s method to solve them, this work will not consider these types of conditions. Because linear systems are sparse, the second step is to compute the nonzero entries of the coefficient matrix and the right-hand-side vector of the linear system. Once the linear systems are prepared, the third step is to solve them with appropriate solvers such as LAPACK [3], UMFPACK [4], MUMPS [5] and HYPRE [6] for the variable values at the next time step. Of course, if some variable values at the next time step can be calculated with fully explicit schemes, no corresponding linear systems for the variables exist, and they must be obtained directly from the explicit expressions. The three steps loop until the desired simulation results are achieved. Thus, the simulation procedure, in reality, involves constructing the linear systems and then solving them, which is a procedure that applies to most applications of computational fluid dynamics. With this philosophy, a general framework to simulate fluid dynamics is suggested in this work. Here, “general” means that the framework is not designed for any specific applications of fluid dynamics. Instead, the code of any specific application is easily and quickly generated by the framework. In addition, practical fluid simulations often come from large-scale projects, such as reservoir simulations ([7–9]), atmosphere simulations [10, 11] and environmental simulations [12, 13], which necessitates the application of parallel computing technology to accelerate the simulations. In parallelization, one key issue is determining ways to perform domain decompositions and then determining how to assemble the information on different processors. In addition, leveraging the threads in a processor to achieve high speeds is the other key issue. In this work, the general framework is parallelized on both levels of threads (OpenMP) and processors (MPI).

From the discussion above, it is surmised that the main operations in this framework include the discretization of the equations and variables, the computation of the coefficients, the assembly of the linear systems, the solving of the systems, etc., and it is assumed that all the operations are done on the fields. Thus, the general framework is in reality the union of all the operations on the field elements, i.e., it is a field-based general framework. A field is a subset of a grid. Some grid elements can be selected to form a field, and these grid elements are also called field elements. For example, if there is a 3D regular grid, the *x*-direction faces, the *y*-direction faces, the *z*-direction faces and the center of all the grid cells are the grid elements. All the *x*-direction faces can form a field, and each of the *x*-direction faces is a field element. Here, the *x*-direction face is defined as the cell face that is perpendicular to the *x*-axis of the coordinate, and the *y*-direction and *z*-direction faces have similar definitions. All the discretized variables (DVs) imposed on a field are called the variable fields, and all the algebraic equations (AEs) imposed on a field are called the equation fields.

Before looking into the details of this work, it is necessary to give a brief review of the state-of-art CFD simulators. Generally, most of the simulators can be run in parallel. However, in a variation of this framework, which can be parallelized with both OpenMP and MPI, some simulators can be parallelized only with either OpenMP or MPI. The simulators parallelized only with OpenMP include HUNS3D [14, 15], ARC3D [16], OVERFLOW [17, 18], FIRE PARADOX [19, 20], etc., and they can be applied in the fields of viscous flow simulations, aerophysics simulations, the design of airfoils and the management of forest fires. This level of parallelization is easy to achieve, and the load of the threads is light [21]. However, its parallel performance when dealing with large-scale simulations is not adequate [22]. Thus, simulators parallelized with MPI are more popular in those conditions. The simulators parallelized only with MPI include ReFESCO [23], elsA [24, 25], TFLO [26], CALC-MP [27], OpenFOAM [28, 29], etc., and they can be used to simulate multiphase viscous flows, the flow behavior in complicated geometries, turbomachinery flows, fluid flows, heat transfers and structural analyses. With the progress of parallelization technology, OpenMP and MPI hybrid simulators have emerged, such as TAU [30], Telemac [31], NetKar [32] and COMSOL [33, 34]. These simulators are trustworthy tools to simulate external flows, environmental flood flows, turbulent flows past a circular cylinder and heat transfers. By hybrid parallelization, both of the advantages of OpenMP and MPI can be leveraged, and the parallel performance is further enhanced. Although the concept of the field operation appears in OpenFOAM, it is applied only in the discretization operation. This work further applies the field concept in the operations of coefficient computing and the assembly of the linear system, which covers more of the simulation procedure. The domain decomposition in OpenFOAM is achieved with the help of MPI, which is more or less the same as the hybrid simulators including the general framework in this paper. However, only some code snippets, such as the loops, are parallelized by OpenMP in the former hybrid simulators, which limits the application of OpenMP and reduces the parallel efficiency. In contrast, the general framework in this work applies OpenMP in the entire coefficient computing routine, which greatly improves the parallel efficiency on the thread level.

This work is organized as follows: Section 1 is an introduction to the general ideas of the framework. Section 2 and Section 3 introduce the discretization library and the coefficient library of this framework, respectively. Section 4 will detail how to construct the linear system, and Section 5 will give the parallelization strategy. Section 6 presents the construction of Masor, which is a matrix acidization simulator developed based on the general framework. The conclusion of this work is in Section 7.

## 2. The discretization library

According to different application conditions, different mathematical models are proposed to simulate the fluids. However, all the models are derived from the same physical laws, i.e., the mass conservation law, the momentum conservation law, the energy conservation law, etc. As a result, the mass conservation equation, the momentum conservation equation, the energy conservation equation, etc., are formed. For this reason, these equations are composed of similar terms, with each of them representing various physical meanings, although they may have different expressions in different models. For example, almost all the mass conservation equations have the divergence term, which describes the mass diffusion in space. The few differences among the mass conservation equations of different models may only lie in the coefficient of the divergence term and the constant term. Moreover, even in the model itself, the divergence term may exist in different equations, for instance, they may exist in the mass conservation equation and the momentum conservation equation. Thus, for any model, the discretization of the equations can be deemed the discretization of the terms. As a result, there exist common discretization patterns that can describe the discretization of most of the equations. This motivates us to develop the discretization library, which is composed of all the common discretization patterns for the equations.

In addition to the fact that the equations need to be discretized, the variables also need to be discretized. In other words, discretization can be classified into two categories, discretization of the equations and discretization of the variables. When the finite difference method is used for discretization, the entire simulation domain is divided into a regular grid, and the discretized equations and variables are imposed either at the center of the cell or on the face of the cell. For example, variables such as pressure, concentration and temperature are often imposed at the center of the cell, and velocity is often imposed on the face of the cell. The upwind scheme and harmonic method can be used to derive the variable values on the face of the cell if they are imposed at the center of the cell. Although there are other discretization schemes, such as finite element methods, and the discretization can be done in a triangular mesh, these schemes will not be discussed in this work.

With this philosophy, discretization can be deemed an operation on the equation and the variable, irrespective of their physical meanings. The equations can be discretized at the center or on the face of the cell, and the variables can also be discretized in that way, so there are various discretization types. The discretization type can be represented by the letters x, y, z and p, which represent discretization on the *x*-direction face, the *y*-direction face, the *z*-direction face and at the center of the cell, respectively. Moreover, when the discretization type of an equation is given, the discretized positions of all the variables of the equation can then be decided. For example, if the equation below is discretized at the center of a cell, which has the discretization type p, then:

$$\frac{\partial p}{\partial t}+\nabla ∙u=0,$$ (1)

The variable *p* is discretized at the center of the cell, and the variable ***u*** is discretized on all the faces of the cell. The discretized positions of a variable in an equation constitute its discretization configuration. For a variable, its variable discretization configuration in an equation may be the same as its variable discretization configuration in a term. For example, the variable discretization configuration of ***u*** in Equation (1) is the same as that in the divergence term. Thus, the name of the variable discretization configuration can be represented by the abbreviations of the name of the term. For example, div, gra, lap and der are the abbreviations of the terms divergence, gradient, Laplace and derivative, respectively, which can be used to represent the corresponding variable discretization configuration of an equation. Different combinations of the discretization type and discretization configuration form different discretization patterns of which the discretization library is composed. In other words, a discretization pattern can be defined by a triple term, as in:

<equation discretization type, variable discretization type, variable discretization configuration>.

In the code of the discretization library, the subroutines recognize the discretization patterns, and the naming rule of the subroutines can be determined in a rational pattern. For example, in the subroutine name “dctz_pxdiv”, “dctz” stands for the meaning of discretization, and “pxdiv” is the triple to represent the discretization pattern. Thus, the subroutine name “dctz_pxdiv” means that the equation is discretized at the center of the cell, and the variable is discretized on the *x*-direction face of the cell. The variable discretization configuration in the equation is the same as that in the divergence term. All the other subroutines obey the same naming rule.

To clarify the use of the discretization library, the discretization of the momentum conservation equation in the matrix acidization simulation can be taken as an example. The equation is shown as below:

$$\rho_{f}\frac{\partial }{\partial t}(\frac{u}{\phi })+\rho_{f}\frac{u}{\phi }∙\nabla \frac{u}{\phi }=-\nabla p-\frac{\mu }{K}u+\nabla ∙\mu \nabla \frac{u}{\phi }-\frac{\rho_{f}F}{\sqrt{K}}|u|u+\rho_{f}g.$$ (2)

**Fig 1:**
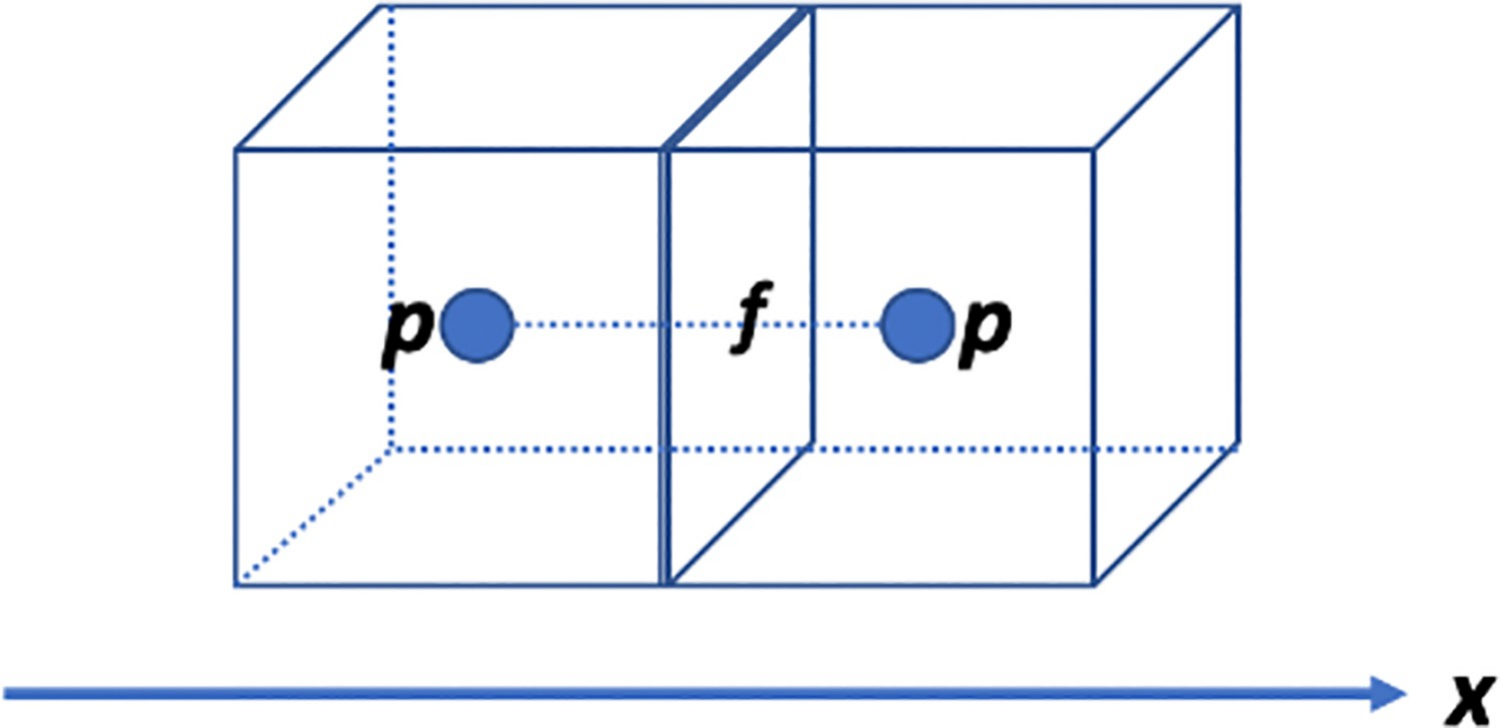
Discretization of the variable *p* in Eq (2). There are two cells in the *x*-direction. Suppose Eq (2) is discretized on the face *f*, and then the variable *p* is discretized at the center of the two cells, which is represented by the two solid points.

**Fig 2:**
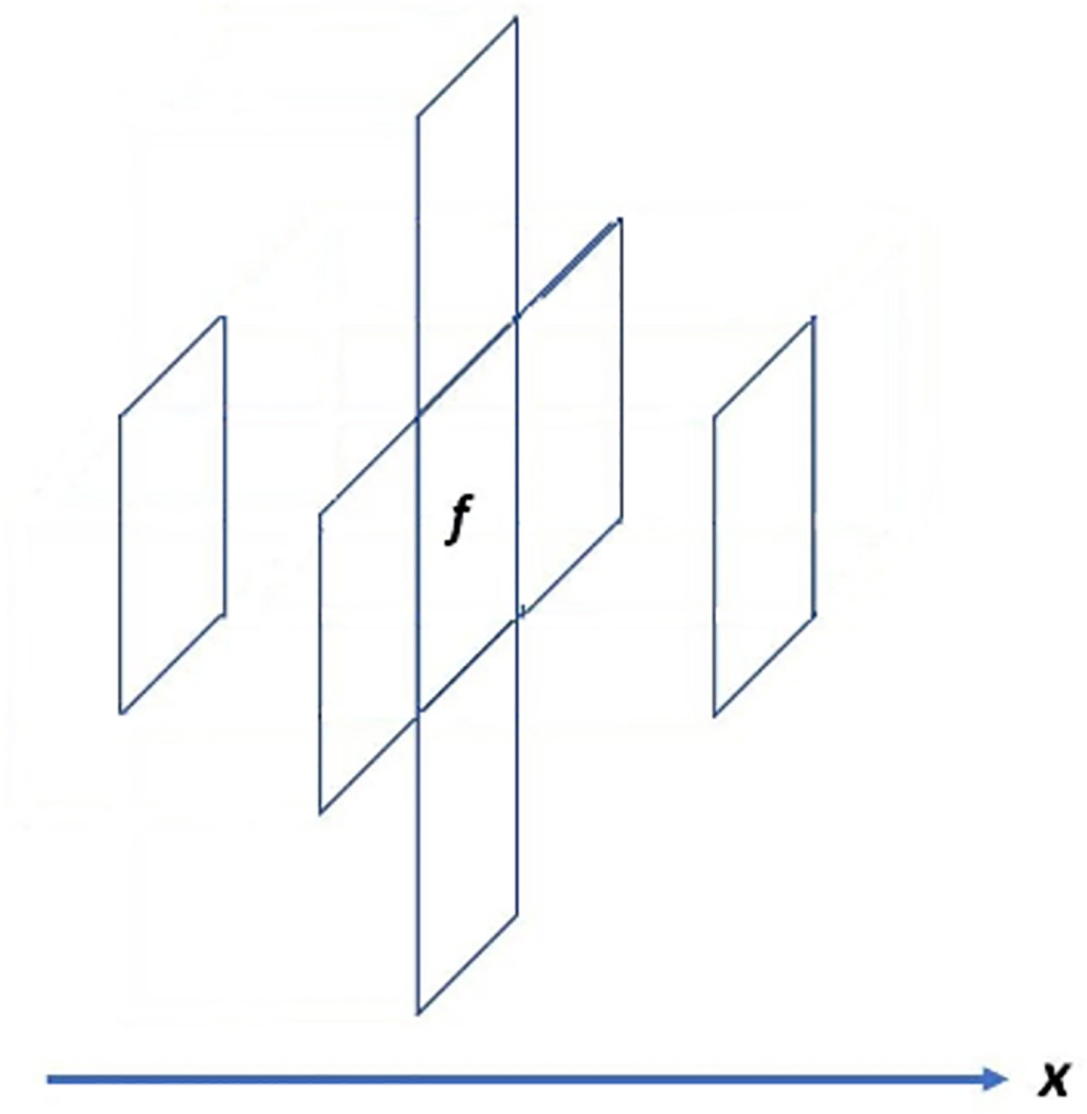
Discretization of the variable *u*_*x*_ in Eq (2). Suppose Eq (2) is discretized on the *x*-direction face *f*, and then the variable *u*_*x*_ is discretized on the face *f* and the six faces adjacent to *f*.

Since the physical meanings of the variables are not important to the discretization of the equation, they will not be given here. The unknown variables of the equation are the scalar *p* and the vector ***u***, and all the other variables are supposed to be known and become the coefficients of the equation. In fact, the equation can be discretized in the *x*-, *y*-, and *z*-directions. When the equation is discretized in the *x*-direction, a discretized equation can be imposed on an *x*-direction face *f*. In this condition, *p* is discretized at the center of the two cells with *f* being their common face, which can be shown by Fig 1. Thus, the subroutine “dctz_xpgra” is used to discretize *p* in the equation. ***u*** is discretized on the *x*-direction face, which makes ***u*** degenerate into its *x*-direction component *u*_*x*_ in the discretized equation. In the first term and the fourth term, *u*_*x*_ is imposed on the face *f*. In the second term, there are two ***u***, where the left ***u*** can be deemed known and the right ***u*** can be deemed unknown. Thus, *u*_*x*_ is imposed on the face *f* and either adjacent face of *f* in the *x*-direction if the upwind scheme is used. The fifth term is in reality a Laplace term, and therefore, *u*_*x*_ is imposed on the face *f* and all its adjacent faces in all three directions, in which there are seven faces in total. The sixth term also has two ***u***, and only the right ***u*** is unknown. Thus, *u*_*x*_ is imposed on the face *f*. From the discussion, it is learned that the variable discretization configuration of *u*_*x*_ in the equation should be the same as that in the Laplace term, which is shown in Fig 2. Thus, the subroutine “dctz_xxlap” is used to discretize *u*_*x*_ in the equation. With the same idea, the subroutine “dctz_yylap” can be used to discretize *u*_*y*_ in the equation, and the subroutine “dctz_zzlap” can be used to discretize *u*_*z*_ in the equation, in which *u*_*y*_ and *u*_*z*_ stand for the *y*-direction and *z*-direction components of ***u***, respectively. More details of the discretization library can be seen in Section 6.

## 3. The coefficient library

The equations in the mathematical model are often partial differential equations (PDEs). After discretization, these PDEs become AEs. Some AEs can be collected and arranged in a certain order to form a linear system. When the unknown variables of the linear system are defined, the coefficients of the unknown variables are the nonzero entries of the coefficient matrix, and the terms without unknown variables form the right-hand-side vector. Thus, the next step is to compute the coefficients and the right-hand-side vector.

Traditionally, the expressions of the coefficients and right-hand-side vector have to be given by hand before obtaining their values. However, this method wastes time when deriving the expressions and is error prone. Thus, a more straightforward method is proposed to avoid these issues. It is noted that an AE in a linear system is in reality a linear AE in this work, whose general expression can be written as:

$$a_{i1}x_{1}+a_{i2}x_{2}+a_{i3}x_{3}+\cdots +a_{in}x_{n}-b_{i}=0,\;i=1,2,\ldots ,n,$$ (3)

if there are *n* variables in the linear system. In Eq (3), *x* stands for the variable, *a* stands for the coefficient of *x* and *b* stands for the right-hand-side vector. With the traditional idea, *a* and *b* are known, and *x* can be calculated from *a* and *b*. However, in the opposite way, if *x* is known, *a* and *b* can also be calculated from *x*. More concretely, if all the *x* are set to be zero, the left-hand side of (3) equals with −*b*_*i*_; if *x*_*j*_ is set to be one, and all the other *x* are set to be zero, the left-hand side of (3) equals with *a*_*ij*_−*b*_*i*_. Since *b*_*i*_ has been known, *a*_*ij*_ can also be known. In this way, the coefficients and right-hand-side vector can be calculated directly from Eq (3) without the step of deriving their expressions.

If it is deemed an iteration that sets *x*_*j*_ as one and the other *x* as zero, *j* = 1,2,…,*n*, and setting all the *x* as zero is also an iteration, a total of *n*+1 iterations are needed to obtain the values of all the coefficients and the right-hand-side vector, with the method above. However, the number of iterations can be reduced further by the improvement below. For the convenience of the following discussion, *b* is put to the right-hand side of (3), and there is:

$$a_{i1}x_{1}+a_{i2}x_{2}+a_{i3}x_{3}+\cdots +a_{in}x_{n}=b_{i},\;i=1,2,\ldots ,n.$$ (4)

For *x*_*j*_, not all the AEs contain it. Those AEs that contain *x*_*j*_ can be called the neighborhood of *x*_*j*_. It is observed that if the neighborhoods of two *x* overlap, the two *x* cannot be set as one simultaneously to achieve their corresponding coefficients in the overlapped equation, since in that condition, the left-hand side of Eq (4) equals the sum of their coefficients. For example, if both *x*_1_ and *x*_2_ are set as one in one of their overlapped equations, the left-hand side of the overlapped equation in the form of Eq (4) equals, *a*_*i*1_+*a*_*i*2_, from which *a*_*i*1_ and *a*_*i*2_ cannot be calculated, respectively. In other words, for all *x* that do not have overlapping neighborhoods, they can be set as one simultaneously to achieve a group of coefficients. In this way, the total number of iterations to achieve all the coefficients and the right-hand-side vector is reduced to the number of groups of *x* that do not have overlapping neighborhoods.

**Fig 3:**
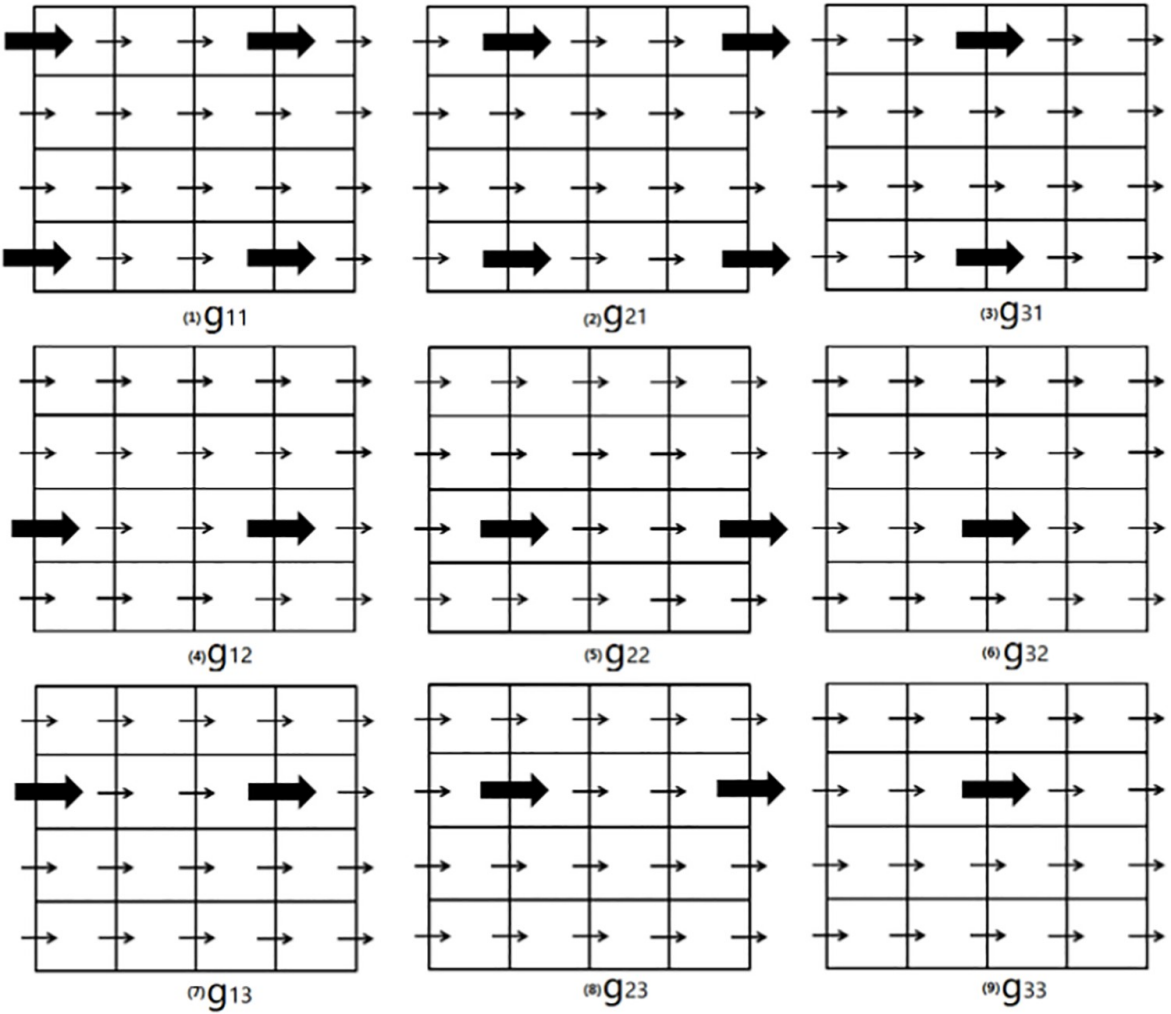
A two-dimensional sketch of the shifting. All the variables are discretized in the *x*-direction, which is represented by arrows. All the DVs are allocated to nine groups, which are represented by *g*_*ij*_, *i*,*j* = 1,2,3. For each group, the arrows allocated to it are in bold. *g*_21_ is derived by shifting *g*_11_ by one position in the positive *x*-direction (the right direction), and *g*_12_ is derived by shifting *g*_11_ by one position in the positive *y*-direction (the up direction). In each group, any pair of adjacent bold arrows has the other two arrows that are not in this group between them.

In fact, there are many ways to divide all the *x* into such groups. However, the way that produces the minimal number of groups is the most interesting, since in that way all the coefficients and right-hand-side vector can be achieved with the minimal number of iterations. For example, it is supposed that a kind of variable is discretized with the pattern “xxlap”. For the DV imposed on the *x*-direction face *f*, its neighborhood is composed of seven equations that are imposed on the face *f* and six adjacent *x*-direction faces of *f*. In that condition, the two DVs separated by the other two DVs can be put in a group *g*_111_. Then, shifting every DV in this group for one position in the *x*-direction generates another group *g*_211_ of this kind. Furthermore, shifting every DV in *g*_211_ for one position in the *x*-direction generates another group *g*_311_. In the same way, it is easy to see that *g*_411_ is the same as *g*_111_; therefore, no further shifting is needed. This kind of shifting can also be done in the *y*-direction and *z*-direction, respectively, which generates twenty-seven groups in total. Fig 3 gives a two-dimensional sketch of this kind of shifting. It is easy to see that the other ways to divide all the DVs will give a larger number of groups. Thus, only twenty-seven iterations are enough to achieve all the coefficients and right-hand-side vectors, which is a great improvement. From the discussion above, a more general conclusion is that the minimal number of groups of a variable depends on the discretization pattern of the variable. The subroutine “genExpField” is used to generate all such groups. In addition to the fact that the number of iterations can be greatly reduced, the improvement method above has the other advantage, which makes the task to achieve the coefficients and right-hand-side vector to be done in parallel. Every DV in a group can be allocated a thread to achieve all its relative coefficients, which is done in parallel on the level of threads and can be done by OpenMP [35, 36].

In the code, all the subroutines to compute the coefficients, the right-hand-side vector and the subroutine “genExpField” comprise the coefficient library. It is observed that in Eq (3), if all *x* are the solutions of the equation, the left-hand side of Eq (3) equals its right-hand side, whose difference is zero. However, if *x* is not the solution of the equation, there is a difference between the left-hand side and right-hand side of the equation, which is termed the residual of the equation. The coefficient computing method above is in fact a procedure to compute the residuals of the equation under different *x*. Thus, the naming rule of the subroutine in the coefficient library can be set as “Resi_equation name_variable name”. Here, “Resi” is the abbreviation of “residual”. For example, the subroutine “Resi_xmom_vx” can be used to compute the coefficients of the *x*-direction velocity variables in the *x*-direction momentum conservation equation of (2).

Since different mathematical models have different PDEs, there are no common subroutines that can be used to compute the coefficients of all the models. Thus, the coefficient library is open, which means that numerous coefficient computing routines have to be developed by the programmer according to different PDEs. However, the coefficient library will stipulate a unified specification for all routines.

## 4. The linear system library

**Fig 4:**
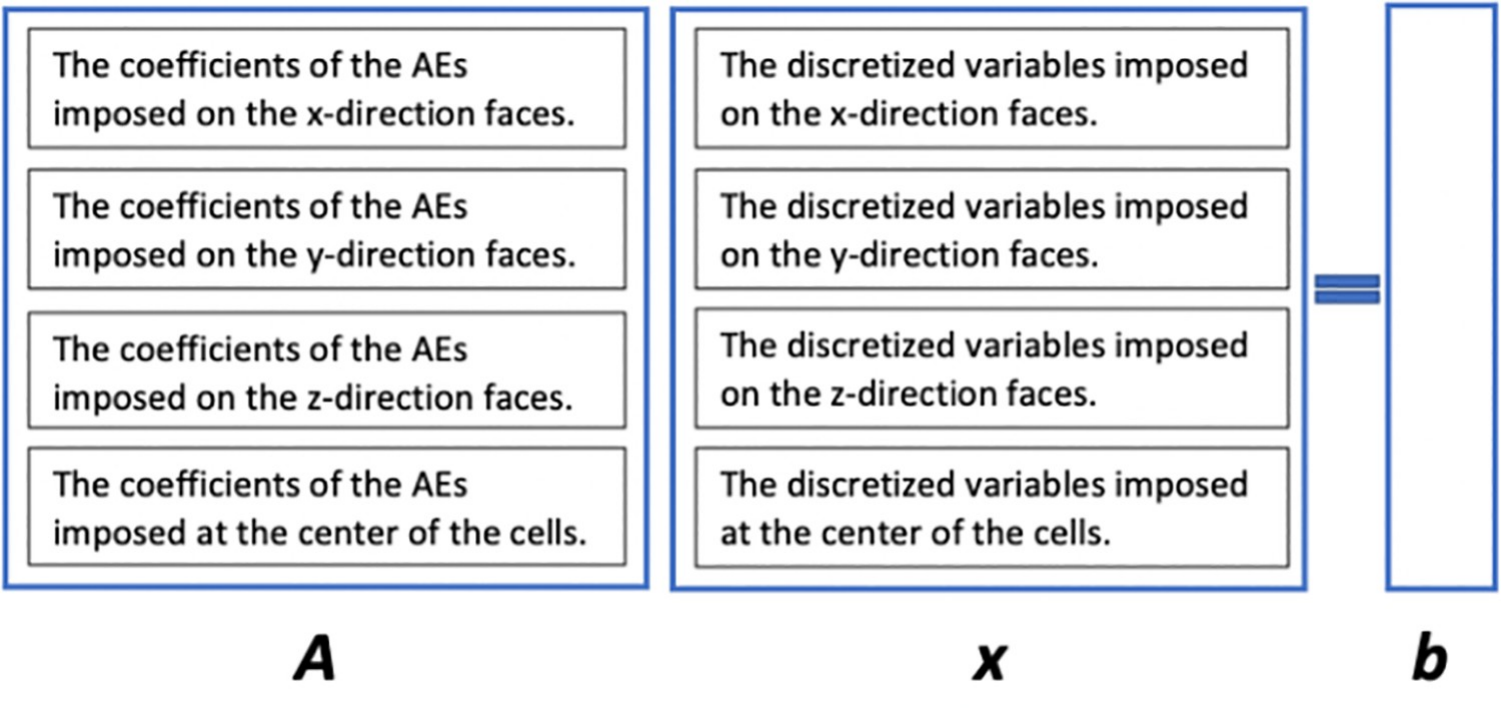
The linear system *Ax* = *b*, where *A* stands for the coefficient matrix, *x* stands for the unknown vector, and *b* stands for the right-hand-side vector.

A linear system can be constructed from one or more PDEs. For example, after discretization, the concentration equation in the DBF model ([37–42]):

$$\frac{\partial (\phi C_{f})}{\partial t}+\nabla ∙(uC_{f})=\nabla ∙(\phi D_{e}∙\nabla C_{f})-k_{c}a_{v}(C_{f}-C_{s}),$$ (5)

which is a PDE, forms a linear system, by which the acid concentration can be achieved. However, another linear system in the DBF model, which is the velocity-pressure linear system, is constructed from two PDEs, the momentum conservation equation and the mass conservation equation. All the PDEs may have different discretization positions, either at the center of the cells or on the faces of the cells. Thus, in the linear system, it is stipulated that the AEs imposed on the *x*-direction faces are put into it first, and then the AEs imposed on the *y*-direction faces, *z*-direction faces and at the center of the cells are put into it one after another. The DVs are put into the unknown vector in the same order. If more than one PDE is discretized on the *x*-direction faces, for example, the order should be that the AEs discretized from the first PDE are put into the linear system first, followed by those from the second PDE, and so on. This rule is the same for the condition of the DVs. Fig 4 shows the linear system *Ax* = *b*, where *A* stands for the coefficient matrix, *x* stands for the unknown vector and *b* stands for the right-hand-side vector.

The row number of an AE in the linear system is called its index. The row number of a DV in the unknown vector of the linear system is called its index, or the column number of a DV in the coefficient matrix is called its index. All the *x*-direction faces in the regular grid form the *x*-direction field, and in the same way, the *y*-direction field and the *z*-direction field can be formed. All the center of the cells form the center field. Every element of the field has its own coordinates in the field. Since all the AEs and DVs are imposed on the field elements, their indices can be derived from the indices of the field elements. For example, if a regular grid has 4, 8, and 16 cells in the *x*-, *y*- and *z*-directions, respectively, the index of the field element with coordinates (2,3,5) in the center field should be:

(4+1)×8×16+4×(8+1)×16+4×8×(16+1)+4×8×(5−1)+4×(3−1)+2=1898. (6)

Here, it is stipulated that all the coordinate entries are natural numbers and that the elements of a field are counted in the order of the *x*-, *y*-, and *z*-directions, which means that the element with the coordinate (1,1,1) is counted first, followed by the element with the coordinate (2,1,1), (3,1,1), and so on. The first three terms of (6) represent the element amounts of the *x*-, *y*-, and *z*-direction fields, respectively. Thus, the AE or DV imposed on this element has an index of 1898 if no more than one PDE or variable is imposed on the element of the center field. The index computing is finished by the subroutine “coordiToGlobalInd”, which will be discussed in more detail later. In fact, the row number of the nonzero entry of the coefficient matrix is the equation index, and the column number is the DV index. Thus, when the two kinds of indices are achieved, the positions of the nonzero entries in the coefficient matrix can be obtained. Since the values of all the nonzero entries of the coefficient matrix and the right-hand-side vector have been known with the help of the coefficient library, the construction of the linear system is completed. The routine “setMatValue” is designed to carry out all these steps.

In the code, the coefficient matrix is stored in many arrays. All the coefficients of a variable in a PDE should be stored in one array. For example, all the coefficients of the variable *p* in Eq (2) should be stored in one array, and all the coefficients of the variables *u*_*x*_, *u*_*y*_ and *u*_*z*_ in Eq (2) should be stored in the other three arrays. If Eq (1) is combined with Eq (2) to form a linear system, another four arrays are needed to store the coefficients of the variables *p*, *u*_*x*_, *u*_*y*_ and *u*_*z*_ in Eq (1). In addition, some auxiliary arrays are introduced to accelerate the position location of the coefficients in the array. An example is given as following. If the array “AxxValues” is used to store the coefficients of *u*_*x*_ in Eq (2), the arrays “AxxRows”, “AxxCols”, “AxxBase” and “AxxNum” are the auxiliary arrays. The first two auxiliary arrays are used to store the row numbers and the column numbers of the coefficients. In that case, AxxRows[i] and AxxCols[i] represent the row number and the column number of AxxValues[i], respectively. Here, “i” is the index of the array entry. It is easy to see that the size of “AxxValues”, “AxxRows” and “AxxCols” equals the number of coefficients of *u*_*x*_. The coefficients of the AE with a smaller index should be put into “AxxValues” before those of the AE with a larger index. Meanwhile, the coefficient of the DV with a smaller index should be put into “AxxValues” before that of the DV with a larger index. In this way, a coefficient can be located in “AxxValues” if its row number and column number are known. The search begins with the head of the array “AxxRows” and stops when it meets the first entry that equals the row number of the searched coefficient. If the index of the entry is “i”, the search then goes to the subarray (from AxxCols[i] to the tail of “AxxCols”) to search for the column number. If the index of the searched column number is “j”, AxxValues[j] is the searched coefficient. The search in “AxxCols” can be finished with a time complexity of *O*(1). However, the search in “AxxRows” begins with the array head, which leads to the time complexity of *O*(*n*). To reduce the time complexity of the search in “AxxRows” to *O*(1), the arrays “AxxBase” and “AxxNum” are introduced. In the two arrays, the index of the entry represents the index of the AE, and therefore, the size of the two arrays equals the number of AEs. AxxBase[i] records the initial position of the coefficients of *u*_*x*_ of the *i*-th AE in “AxxValues”, and AxxNum[i] records the number of coefficients of *u*_*x*_ of the *i*-th AE. With the help of the two arrays, the search in “AxxRows”, “AxxCols” and “AxxValues” can begin with the index AxxBase[i] for the *i*-th AE and stop at the index AxxBase[i]+AxxNum[i]. As a result, the search for a coefficient in “AxxValues” can be finished in a time complexity of *O*(1).

**Fig 5:**
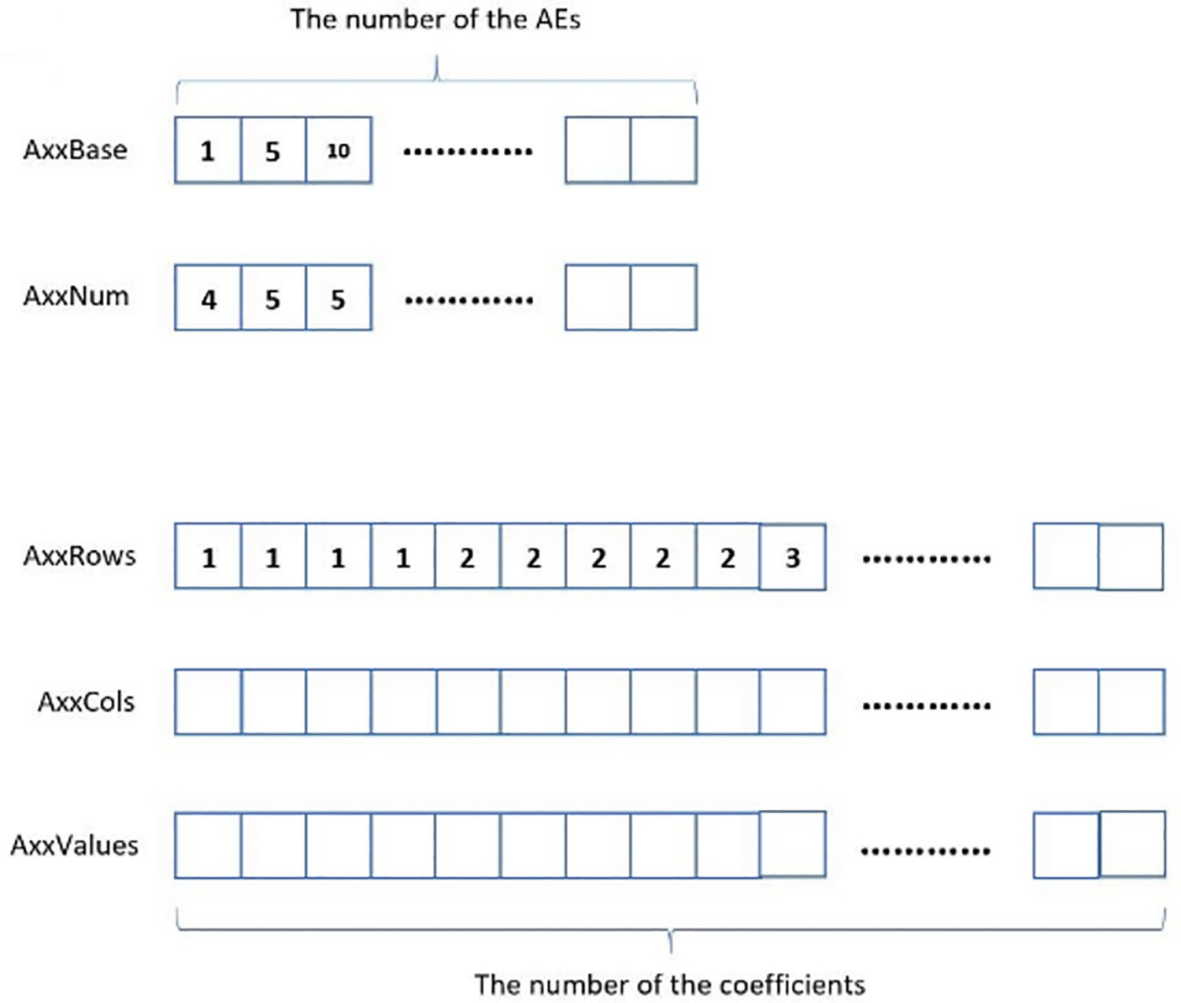
The five arrays help store the coefficients of *u*_*x*_ in Eq (2).

The arrays can be shown in Fig 5. When discretizing Eq (2) in the *x*-direction, the first AE of Eq (2) is located on the *x*-direction face with coordinates (1,1,1), and it has four coefficients of *u*_*x*_. Thus, if the index of the AE is one, the first four entries of “AxxRows” have values of one, the first four entries of “AxxCols” store the column numbers of the coefficients and the first four entries of “AxxValues” store the four coefficients. Since the coefficients of *u*_*x*_ of the first AE are stored from AxxValues[1] to AxxValues[4], there are AxxBase[1] = 1 and AxxNum[1] = 4. In the same way, the second AE is located on the *x*-direction face with coordinates (2,1,1), and it has five coefficients of *u*_*x*_. Thus, AxxBase[2] = 5, and AxxNum[2] = 5.

## 5. Parallelization

**Fig 6:**
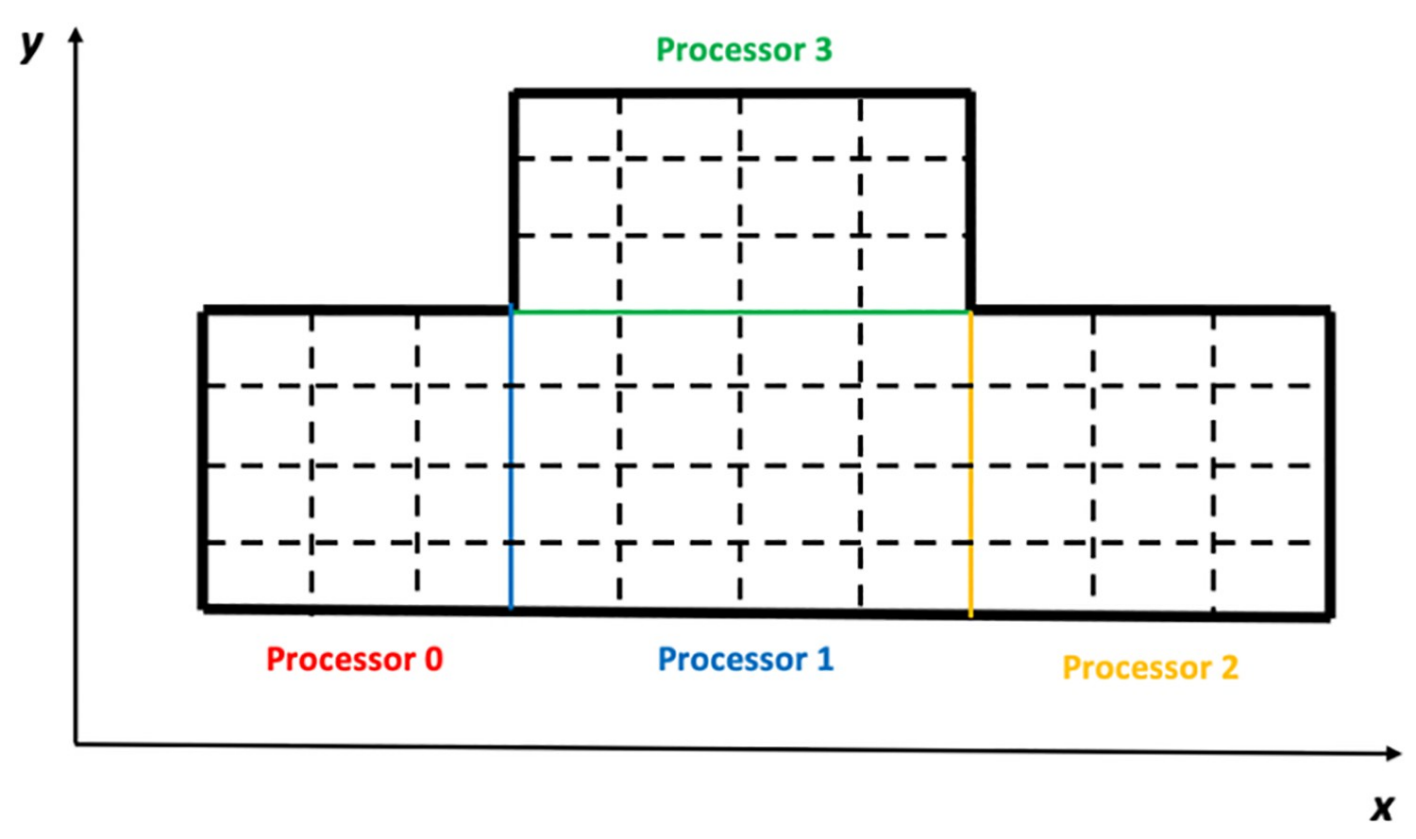
A two-dimensional irregular domain is divided into four regular subdomains. The interfaces of the four subdomains are represented by the three thin and solid lines with three different colors. The blue line is allocated to Processor 1, the yellow line is allocated to Processor 2, and the green line is allocated to Processor 3.

The configuration of the real domain may be irregular. However, it can be decomposed into many smaller cuboids that are then discretized by regular grids. The cuboids are also called subdomains. In the same way, the entire field is also decomposed into many smaller fields that are called local fields. The two boundaries of the subdomain in the *x*-direction are called its left and right boundary, respectively, and the *x*-direction coordinate of the left boundary should be smaller than that of the right boundary. In the same way, the two boundaries of the subdomain in the *y*-direction are called its down and up boundaries, respectively, and the two boundaries of the subdomain in the *z*-direction are called its front and back boundaries, respectively. Fig 6 shows a two-dimensional sketch of a domain that is surrounded by thick and solid lines. It is easy to see that the domain is irregular. However, it can be divided into four regular subdomains. The three thin and solid lines inside the domain are the interfaces of the four subdomains. The thin and dashed lines represent the regular grids of the subdomains.

The operations relative to one subdomain should be finished only by one processor, while one processor should process the operations of only one subdomain. As mentioned above, AEs and DVs are imposed either at the center of the cells or on the faces of the cells. Thus, the operations relative to a subdomain include the coefficient computation of the AEs and putting the coefficients into the linear system. The operations of the AEs imposed at the center of the cells are automatically finished by the processor allocated to the subdomain. However, to discuss the operations of the AEs imposed on the faces of the cells, the arrangement of the processors has to be given. Generally, all processors form a regular grid. If the processors are counted in the order of the *x*-, *y*- and *z*-directions, the index of a processor can be obtained with the same method mentioned above. Some faces of the cells are the interfaces between two subdomains, and therefore, it is stipulated that the AEs imposed on the interfaces are allocated to the processor with a smaller index. The AEs imposed on the other faces are automatically processed by the processors allocated to their subdomains. With this philosophy, the four subdomains in Fig 6 are allocated to four processors, and the arrangement of the four processors can also be seen in the figure. The three interface lines are in different colors, which means that the blue line is allocated to Processor 1, the yellow line is allocated to Processor 2 and the green line is allocated to Processor 3. The numbers “1”, “2” and “3” are called the IDs of the processors. It is noted that the size of the subdomains is more or less the same, which guarantees the load balance of the processors. The domain decomposition strategy on the level of processors can be implemented by MPI [43, 44]. As mentioned above, the coefficient computing in Section 3 is parallelized on the level of threads and can be implemented by OpenMP; therefore, the parallelization of the general framework can be implemented by both OpenMP and MPI.

**Fig 7:**
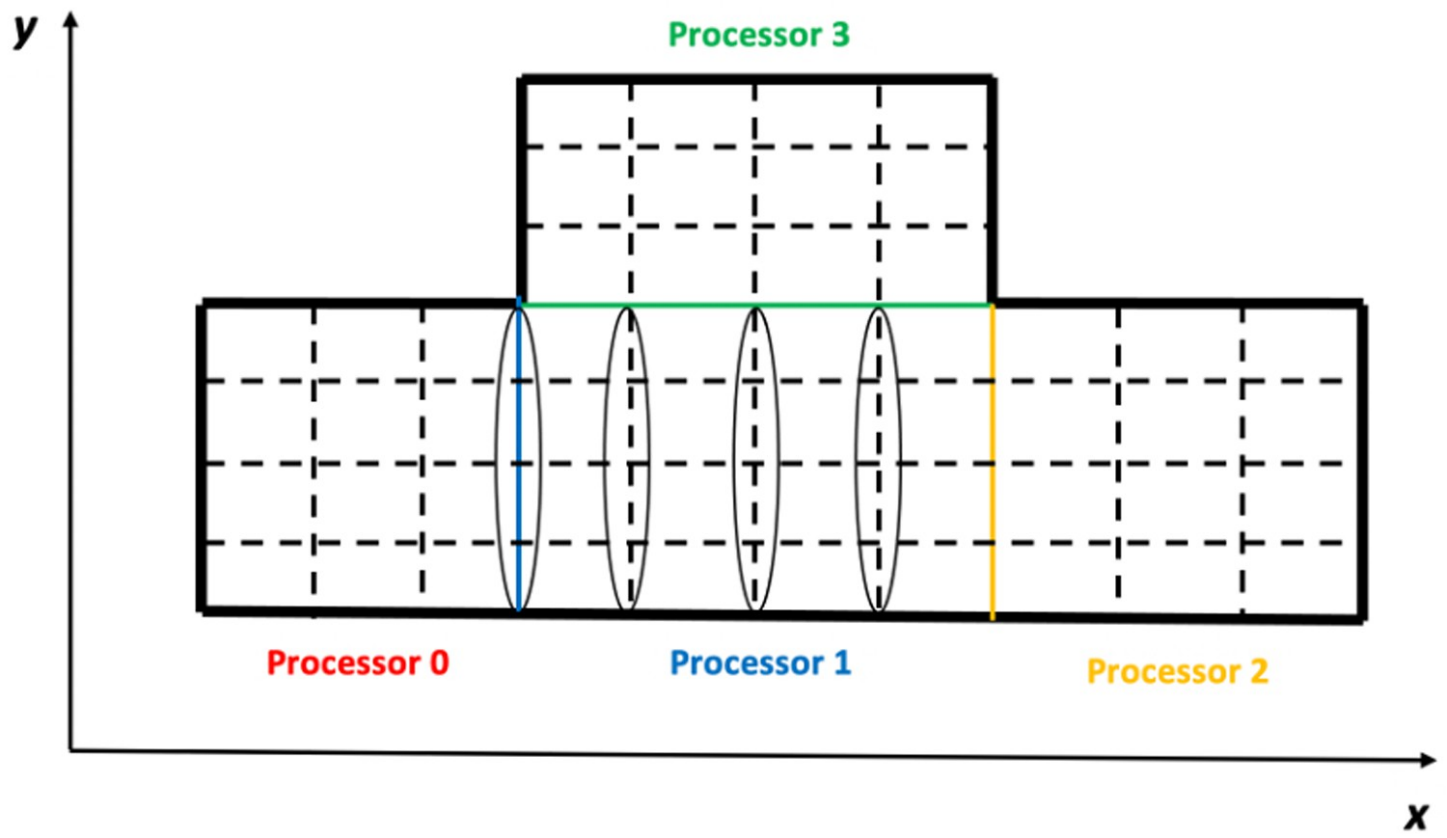
For the discretization pattern “xxlap”, the positions of the AEs on the subdomain of Processor 1 are surrounded by the four circles.

**Fig 8:**
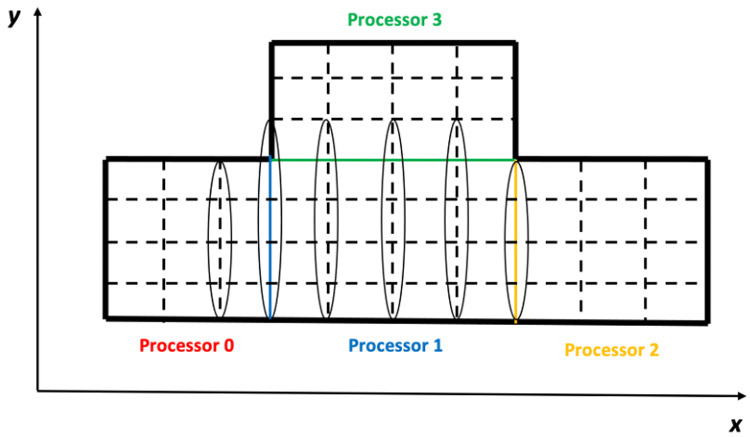
For the discretization pattern “xxlap”, the positions of the DVs of the AEs on the subdomain of Processor 1 are surrounded by six circles.

When computing the coefficients of an AE in the boundary region of a subdomain, some of its DVs may be imposed on the field elements belonging to the neighboring subdomains. Under this condition, the field of this subdomain has to be expanded for these DVs to include the relative field elements of the neighboring subdomains. For the various kinds of DVs, the configurations of the expanded fields are different, which is determined by the discretization patterns. For example, for the discretization pattern “xxlap”, its AEs are imposed on the *x*-direction field of the subdomain, except for the *x*-direction faces on the right boundary. Fig 7 shows the positions of the AEs on the subdomain of Processor 1 in Fig 6, which are surrounded by the four circles. Given this, the DVs of the discretization pattern should be imposed on all the elements of the *x*-direction field of this subdomain and on all the elements of the *x*-direction field that are adjacent to this subdomain, except those adjacent to the right boundary of this subdomain. Here, the elements of the *x*-direction field that are adjacent to this subdomain are defined as the elements; when they are shifted for one position in any of the three directions, they will become the elements of the *x*-direction field of this subdomain. Fig 8 shows the positions of the DVs of the AEs on the subdomain of Processor 1 in Fig 6, which are surrounded by the six circles.

To compute the coefficients of the DVs imposed on the field elements of the neighboring subdomains, some values of the boundary areas of the neighboring subdomains have to be transferred to this subdomain; in the other direction, some values of the boundary area of this subdomain should also be transferred to its neighboring subdomains. These transmissions constitute the communication cost of the processors, which is also the reason that the linear speedup of the parallel code deteriorates with the increase in the number of processors.

In addition, Section 4 gives the indices of the AEs and DVs. The index in a subdomain is called the local index, and the index in the entire domain is called the global index. It is easy to know that the indices computed from Section 4 are local indices. However, in the parallel code, the indices in a linear system should be global indices. Thus, before constructing the linear system, the local indices have to be changed to global indices. If the indices of the processors begin with zero, it is stipulated that the AEs processed by Processor 0 are arranged in the linear system first, followed by those processed by Processor 1, Processor 2 and so on. As a result, the global index of an AE or DV in a subdomain can be achieved by adding the number of local indices of all the former processors to its local index.

This way to construct the linear system can be easily used by many popular solvers. These solvers provide the interfaces and routines to fill the nonzero entries of the coefficient matrix and right-hand-side vector as long as the locations of the nonzero entries are given. The location of a nonzero entry of the coefficient matrix is in reality an ordered pair <row number, column number>. Fortunately, the row number is the AE index, and the column number is the index of the DV. Both of them have been given by the construction method.

## 6. Matrix acidization simulator

### 6.1 The mathematical model

A matrix acidization simulator (Masor) based on the general framework suggested above is developed. Masor simulates a reservoir application in which an acid flow is injected into the matrix to produce an oil production channel. Thus, numerical simulation is a topic of computational fluid dynamics. When matrix acidization is simulated under a constant temperature, the energy conservation law is not considered. Thus, based on the mass conservation law and the momentum conservation law, a mathematical model of matrix acidization can be derived, which is the DBF model. In this model, the mass conservation equation is shown as:

$$\frac{\partial \phi }{\partial t}+\nabla ∙u=0.$$ (7)

Eq (2) is the momentum conservation equation, and Eq (5) is the concentration equation. In particular, the momentum conservation equations in the *x*-direction, the *y*-direction and the *z*-direction are called the *x*-momentum equation, the *y*-momentum equation and the *z*-momentum equation, respectively. The meanings of all the variables in these equations are shown in Table 1. Although there are the other equations in this model, they will not be given in this section, since the purpose here is to give an example of the application of the general framework instead of an investigation of the model. The decoupled scheme [45] is used to solve the model. In this scheme, some changes have to be made to the mass conservation equation and the momentum conservation equation. After that, the mass conservation equation is expressed as:

$$\frac{\partial \phi }{\partial t}+\nabla ∙{\left(\frac{\mu }{K}+\frac{\rho_{f}F}{\sqrt{K}}|u^{n}|\right)}^{-1}(-\nabla p^{n+1}+\rho_{f}g)=0,$$ (8)

which is an elliptic PDE of the unknown variable *p*^*n*+1^. Here, the superscripts represent the time steps. The momentum conservation equation is shown as:

$$\frac{\rho_{f}}{\Delta t}\left(\frac{u^{n+1}}{\phi^{n+1}}-\frac{u^{n}}{\phi^{n}}\right)+\left(\frac{\mu }{K}+\frac{\rho_{f}F}{\sqrt{K}}|u^{n}|\right)(u^{n+1}-u_{D}^{n+1})+\rho_{f}\nabla ∙\left(\frac{u^{n}}{\phi^{n}}⨂\frac{u^{n+1}}{\phi^{n+1}}\right)-\mu \nabla^{2}\frac{u^{n+1}}{\phi^{n+1}}=0,$$ (9)

in which ***u***^*n*+1^ is the unknown variable. It is noted that ***u***_*D*_ is an intermediate variable, which can be expressed as:

$$u_{D}^{n+1}={\left(\frac{\mu }{K}+\frac{\rho_{f}F}{\sqrt{K}}|u^{n}|\right)}^{-1}\left(-\nabla p^{n+1}+\rho_{f}g\right).$$ (10)

**Table 1:** The meanings of the notations.

Notation	Meaning	Notation	Meaning
*p*	pressure	*t*	time
*μ*	fluid viscosity	***g***	gravity vector
*K*	permeability value	*C*_*f*_	cup-mixing concentration of the acid
***u***	velocity vector	***D***_***e***_	effective dispersion tensor
*ϕ*	porosity	*k*_*c*_	local mass-transfer coefficient
*ρ*_*f*_	mass density of the fluid	*a*_*v*_	interfacial surface area per unit volume
*F*	Forchheimer coefficient	*C*_*s*_	concentration of the acid at the fluid-solid interface

### 6.2 The structure of the code

The configuration of the matrix domain to be acidized could be complicated. However, by using the domain decomposition strategy in Section 5, a complicated domain can be divided into a series of subdomains of cuboids, and each of them is allocated to each processor. With the finite difference method on the regular grid, the discretization library is used by each processor to discretize all the equations and variables on its field elements. After that, the coefficient library is used to compute the coefficients of all the AEs. Then, the linear system library is applied, and three linear systems are formed, one is the pressure linear system for solving the pressure, which is composed of the AEs discretized from Eq (8); one is the velocity linear system for solving the velocity, which is composed of the AEs discretized from Eq (9); and the other is the concentration linear system for solving the concentration, which is composed of the AEs discretized from Eq (5).

**Fig 9:**
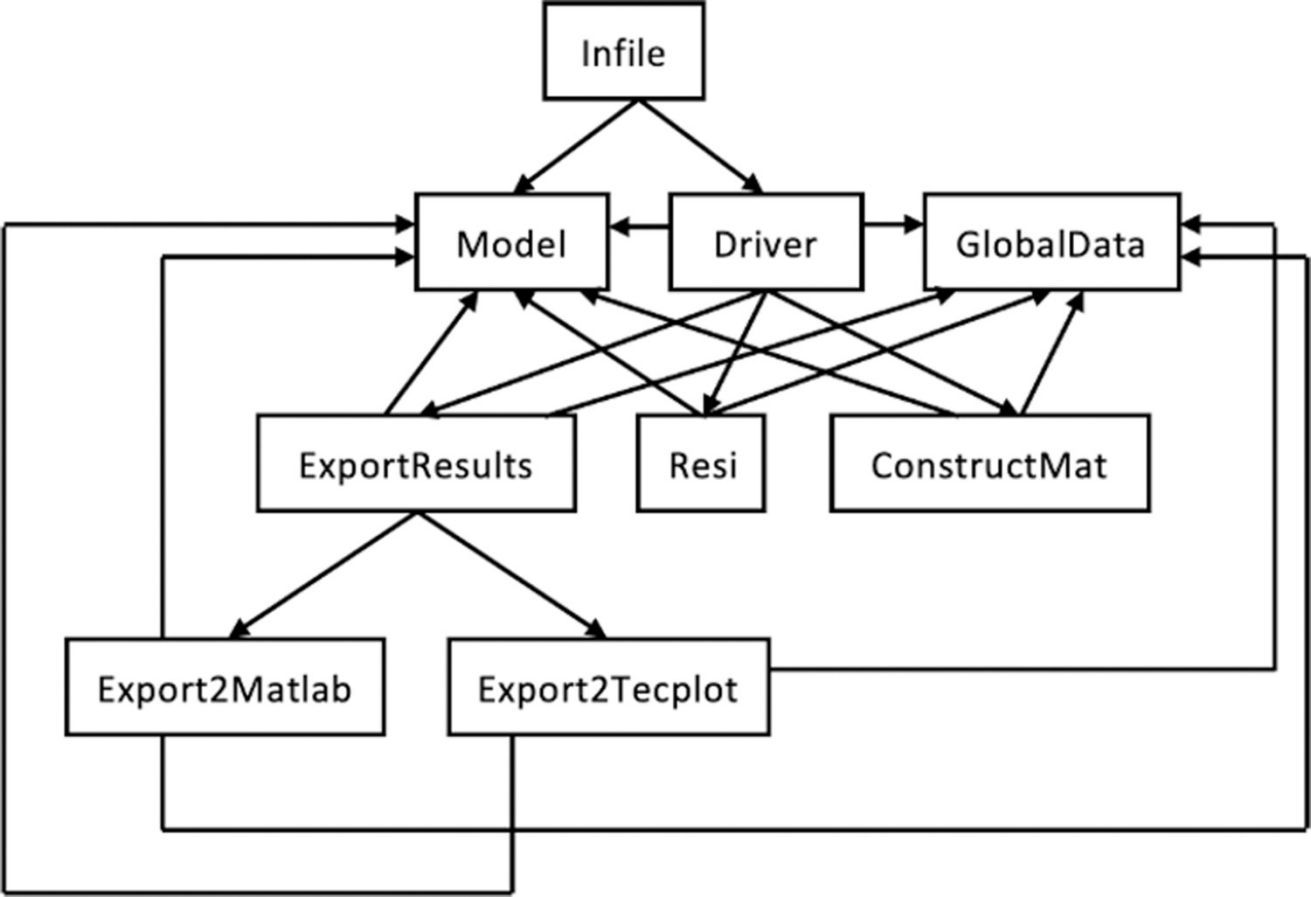
The nine modules in the code of the decoupled scheme of matrix acidization. The arrow represents the calling relationship between two modules, i.e., the module at the tail of the arrow will call the module at the head of the arrow.

The parallel code of the decoupled scheme of matrix acidization is written in FORTRAN 90, OpenMP and MPI, and there are nine modules in this code. The libraries in the general framework are also implemented in FORTRAN 90. Thus, the modules can directly call the subroutines of the general framework to finish the simulation. The nine modules are shown in Fig 9. The user can set the experimental parameters in the module “Infile”. The module “Model” stipulates the variables and parameters that must be set by the users before running the code. The module “GlobalData” defines a series of global data that will be used by the entire code. The module “Driver” defines the flow chart of the code and therefore controls its running. After the experimental parameters are set in the module “Infile”, “Infile” will call the modules “Model” and “Driver” to begin the running of the code. In the running, the module “Driver” will call the modules “Model”, “GlobalData”, “Resi”, “ConstructMat” and “ExportResults” to finish various tasks. Among them, the module “Resi” is designed to compute the coefficients of the AEs, and therefore, it will call many subroutines in the coefficient library. The module “ConstructMat” is used to discretize the PDEs and construct the linear system. Thus, it will call the subroutines in the discretization library and the linear system library. The module “ExportResults” is responsible for outputting the interesting results in either of the two formats, MATLAB and Tecplot, which are implemented by calling the two modules “Export2MATLAB” and “Export2Tecplot”, respectively. It is emphasized that except for the module “Infile”, all the other modules need to call the two modules “Model” and “GlobalData” to finish their tasks.

### 6.3 The module “Driver”

Since the modules “Driver”, “Resi” and “ConstructMat” have a close relationship with the libraries of the general framework, their details will be discussed in the following sections. The study of the other modules will not be given.

**Fig 10:**
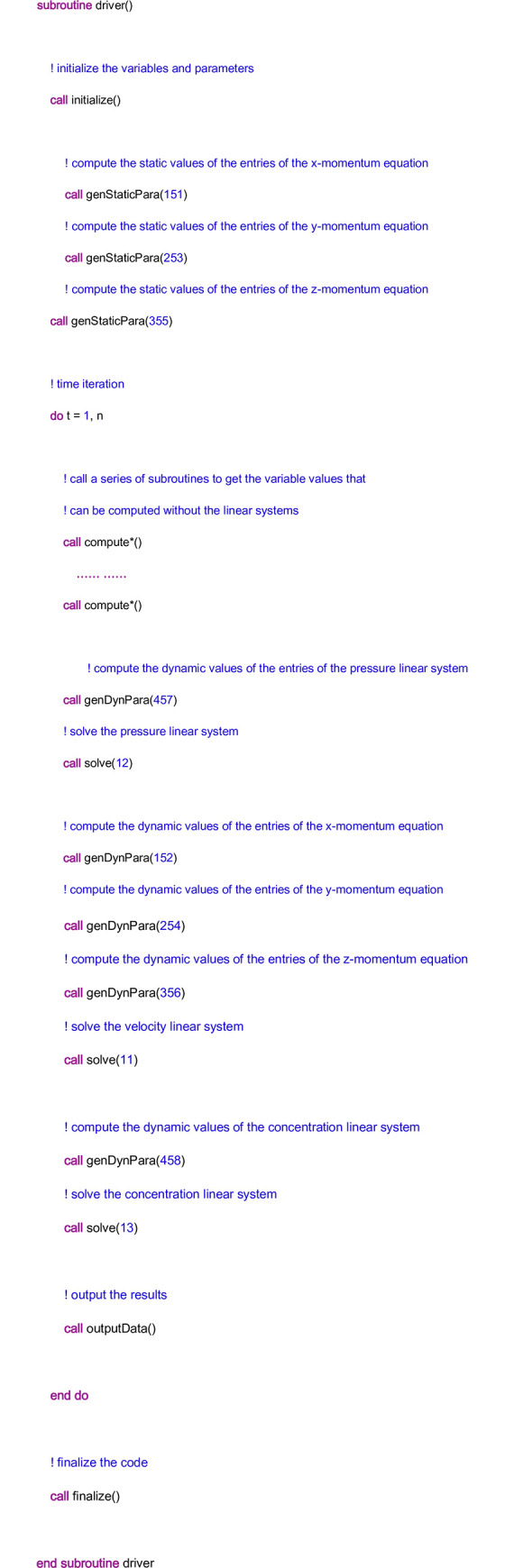
The pseudocode of the subroutine “driver” in the module “Driver”.

In the module “Driver”, there is a subroutine also called “driver”, in which a series of calls are defined. Its pseudocode is given in Fig 10. From the figure, it can be seen that the main task of the subroutine “driver” is to control the running of the code. First, initialization is performed to initialize a series of variables and parameters, such as the initial pressure and the initial porosity in the matrix. Moreover, it is observed that the nonzero entry of the linear system may be composed of many terms. The values of some of the terms will not change with time, and therefore, they are called static terms. On the other hand, if the values of the terms change with time, they are called dynamic terms. For example, on the acid injection boundary of the matrix, the injection velocity is prescribed, and therefore Eq (9) deteriorates to:

$$u=u_{B},$$ (11)

in which ***u***_*B*_ is the prescribed injection velocity. In this condition, ***u***_*B*_ will not change with time, and therefore, it is a static term. Due to the static characteristics of the terms, it is not necessary to update their values at each iteration. Instead, their values should be calculated one time before the time iterations, which is implemented by the calling of the subroutine “genStaticPara”. It is noted that there are no static terms in the entries of the pressure linear system and the concentration linear system. Then, the time iterations begin. The values of some variables can be obtained directly from the explicit formulae, which is done by the calling of the subroutines “compute*”. Here, “*” is a wildcard and represents the name of the variable. After that, it comes to the processing of the three linear systems. With the decoupled scheme, the pressure at time step *n*+1, i.e., *p*^*n*+1^, should be computed from the pressure linear system first, and then with *p*^*n*+1^; the intermediate variable $u_{D}^{n+1}$ can be obtained from Eq (10), which is then used to compute the velocity at time step *n*+1, i.e., ***u***^*n*+1^. With *p*^*n*+1^ and ***u***^*n*+1^, the concentration linear system can be used to compute the concentration at the time step *n*+1, i.e., $C_{f}^{n+1}$. Thus, in the subroutine “driver”, the pressure linear system is processed first, followed by the processing of the velocity linear system and the concentration linear system. The subroutine “genDynPara” helps to compute the values of the dynamic terms, and the subroutine “solve” helps to solve the linear system. Afterward, the results at each iteration are output by the subroutine “outputData”. Finally, the subroutine “finalize” is called to perform some operations to finish the running of the program, such as freeing the memory of the variables. It is noted that the input variable of the subroutines “genStaticPara” and “genDynPara” is “fi_kind”, and the input variable of the subroutine “solve” is “mat_kind”, which represents a kind of coefficient matrix. More details of the two input variables will be given later. Since the other subroutines of the module “Driver” have little relationship with the general framework, they are not discussed.

### 6.4 The module “Resi”

**Fig 11:**
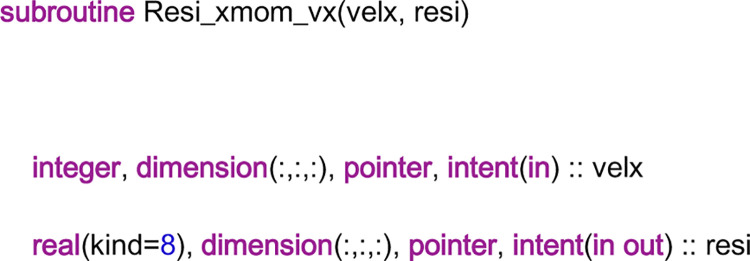
The head part of the code of the subroutine “Resi_xmom_vx”.

The module “Resi” is used to compute the coefficients of the AEs, and therefore, it needs to call the subroutines in the coefficient library. It is known that these coefficients can be derived from computing the residuals of the AEs. To see how to do that, the subroutine “Resi_xmom_vx” is taken as an example. Fig 11 is the head part of the code of the subroutine. The name of the subroutine hints that it will compute the coefficients of the AEs derived from the *x*-momentum equation of Eq (9). The unknown variable of the *x*-momentum equation is the *x*-direction velocity. Thus, the input variable “velx” represents the field of the *x*-direction velocity, which is a three-dimensional array. The input variable “resi” is also a three-dimensional array, which is used to store the residuals of the AEs. Considering that the configuration of the *x*-direction velocity field may be irregular, some entries of “velx” may not be in the *x*-direction velocity field. For example, in Fig 8, “velx” is a 6 × 5 array on Processor 1. However, the entries at the upper left corner and the upper right corner are not in the *x*-direction velocity field. The input variable “resi” is also a three-dimensional array, which is used to store the residuals of the AEs. If the AEs are imposed on Processor 1 of Fig 8, “resi” is a 4 × 4 array.

As mentioned above, the field of the *x*-direction velocity can be divided into 27 groups. The *x*-direction velocities in the same group can be set as one simultaneously to achieve a group of coefficients. Thus, the subroutine “Resi_xmom_vx” will be called 27 times to obtain all the coefficients. For each time, the input variable “velx” is set to represent one group, i.e., the entries of “velx” that represent the *x*-direction velocities in this group are set as one, and the other entries are set as zero. Then, after computation, the entries of “resi” that represent the AEs containing the *x*-direction velocities in this group are the corresponding residuals, which can be sent to the discretization library to fill them in the coefficient matrix of the velocity linear system. Moreover, the subroutine “Resi_xmom_vx” will be called one more time to obtain the right-hand-side vector of the velocity linear system. At this time, all the entries of “velx” are set as zero. The other subroutines in the module “Resi” will not be introduced here.

### 6.5 The module “ConstructMat”

In the module “ConstructMat”, the subroutines “dctz_xxlap”, “dctz_yylap” and “dctz_zzlap” in the discretization library are called to discretize ***u*** in the *x*-momentum equation, the *y*-momentum equation and the *z*-momentum equation, respectively. Moreover, the subroutine “dctz_pplap7” is used to discretize *p* in the pressure equation, and the subroutine “dctz_pplap19” is used to discretize *C*_*f*_ in the concentration equation. From the name of the two subroutines, it can be infered that both the variable discretization configuration of *p* in the pressure equation and that of *C*_*f*_ in the concentration equation are the same as in the Laplace term. However, due to the difference in the discretization schemes of the Laplace term in the two equations, the variable discretization configurations of *p* and *C*_*f*_ are slightly different and are distinguished by the two numbers “7” and “19” in the name of the two subroutines. From Eq (8), it can be seen that when the pressure equation is discretized at the center of a cell, the pressure *p* can be discretized at the center of the six cells adjacent to the cell and at the center of the cell itself, which is the reason that there is the number “7” in the subroutine name “dctz_pplap7”. However, when the concentration equation is discretized at the center of a cell, due to the existence of the tensor ***D***_*e*_ in the Laplace term of Eq (5), the concentration *C*_*f*_ should be discretized at the center of more than seven cells. In a grid of 3 × 3 × 3 cells, if the concentration equation is discretized at the center of the grid, the concentration *C*_*f*_ should be discretized at the center of all the cells of the grid, except the eight cells at the corners of the grid. Thus, the number “19” appears in the name of the subroutine “dctz_pplap19”.

**Fig 12:**
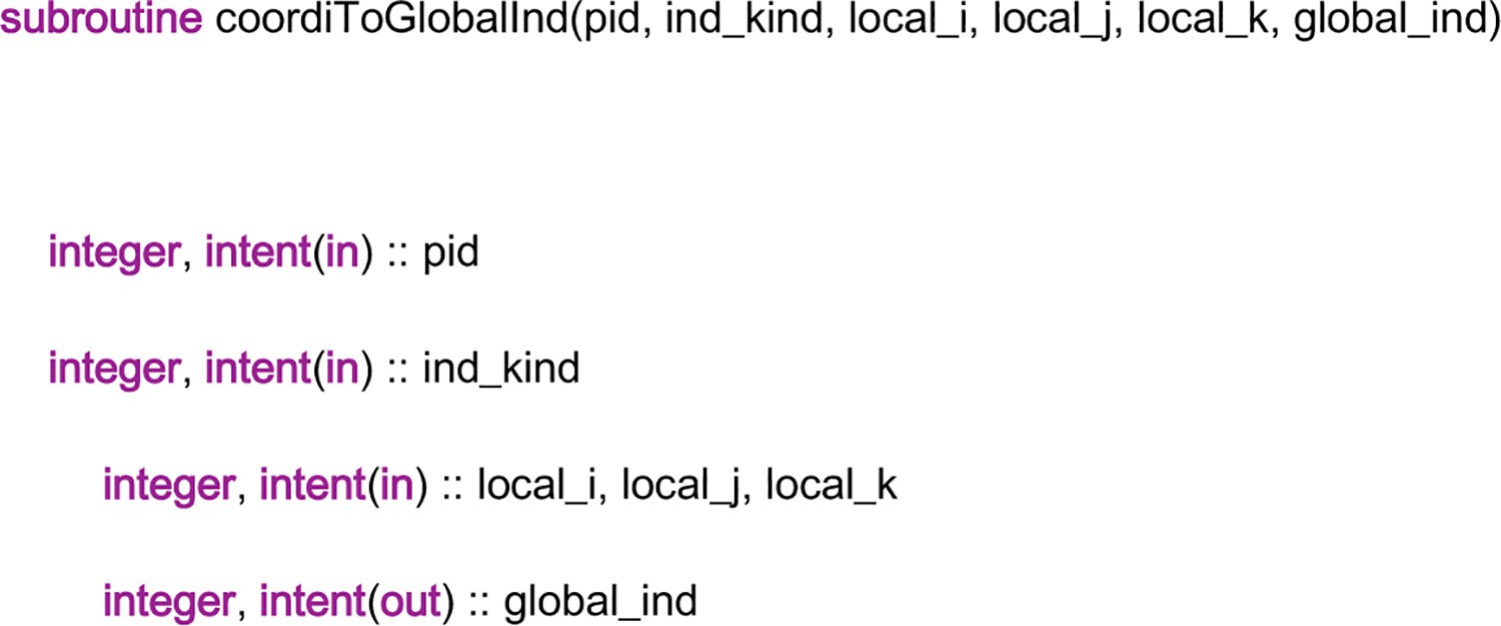
The head part of the code of the subroutine “coordiToGlobalInd”.

In the linear system library, two subroutines, “coordiToGlobalInd” and “setMatValue”, are called by all the subroutines “dctz_*”, to finish the discretization procedure. “*” is a wildcard. The task of the subroutine “coordiToGlobalInd” is to convert the coordinate of a field element in a local field to its index in the global linear system. The head part of the code of the subroutine “coordiToGlobalInd” is given in Fig 12. From the figure, it can be seen that there are six input variables in the parameter list. Among them, “pid” is the processor ID, which indicates the local field on which the field element lies. “local_i”, “local_j” and “local_k” form the coordinates of the field element in the local field, and “global_ind” is the index of the field element in the global linear system. The input variable “ind_kind” is a three-digit integer. The first digit represents the field element type. There are four kinds of field elements, the *x*-direction face, the *y*-direction face, the *z*-direction face and the center of a cell, which are represented by the digits “1”, “2”, “3” and “4”, respectively. The last two digits represent the type of coefficient matrix. “11” stands for the coefficient matrix of the velocity linear system, “12” stands for the coefficient matrix of the pressure linear system, and “13” stands for the coefficient matrix of the concentration linear system. Then, according to “pid”, “ind_kind” and the coordinate, the “global_ind” of a field element can be computed with the methods in Section 4 and Section 5.

**Fig 13:**
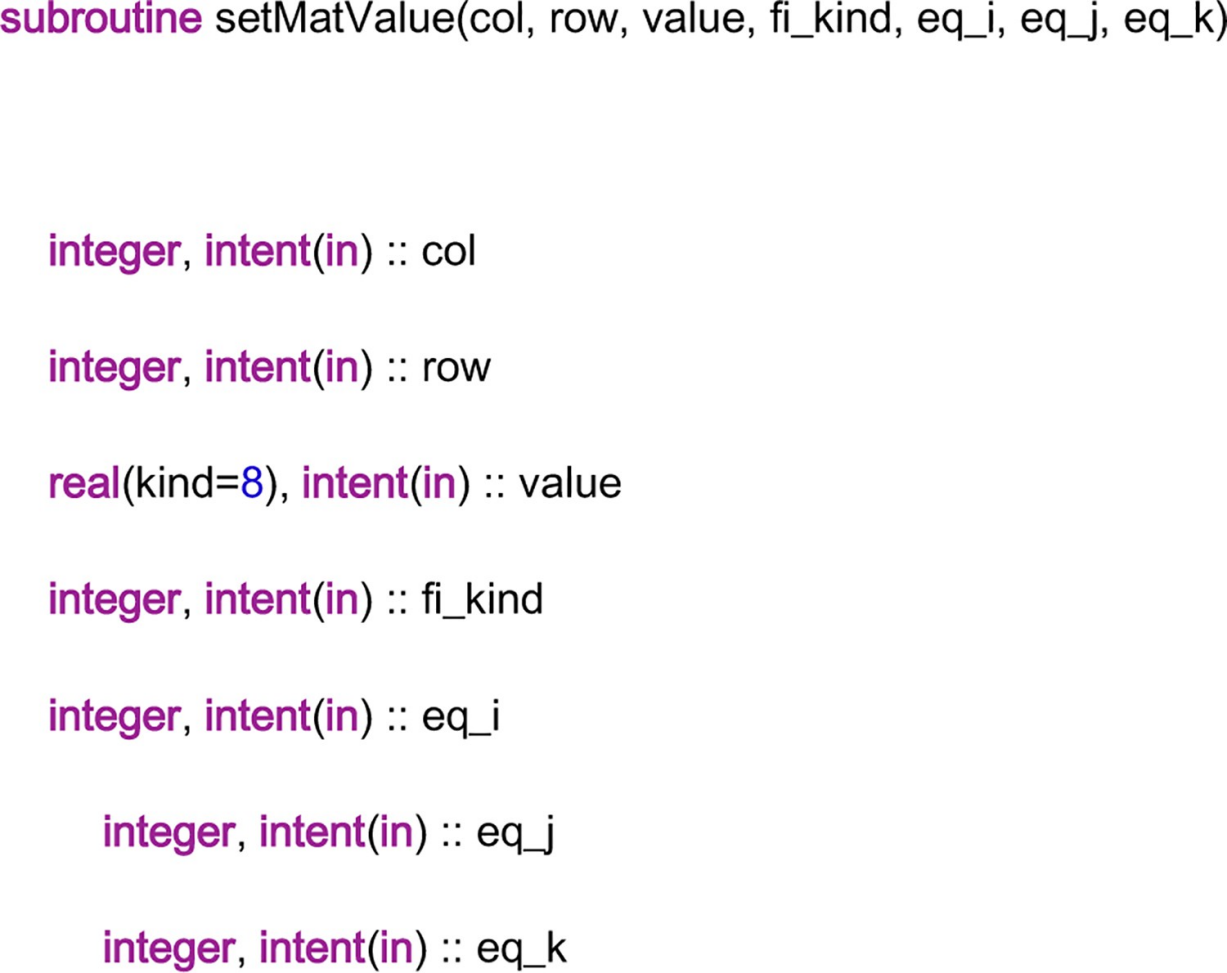
The head part of the code of the subroutine “setMatValue”.

From the discussions in Section 4, it is understood that if a DV is imposed on this field element, the index of the DV in the global linear system is the index of this field element; if an AE is imposed on this field element, the index of the AE in the global linear system is also the index of this field element. Furthermore, the index of an AE is the row number of the AE in the coefficient matrix, and the index of a DV is the column number of the DV in the coefficient matrix. Thus, by the two indices, the position of a nonzero entry of the coefficient matrix, i.e., the position of a residual, can be located. Then, the subroutine “setMatValue” is used to fill the residual in its position of the linear system. The head part of the code of the subroutine “setMatValue” is shown in Fig 13. There are six input variables in the parameter list. The input variable “col” represents the column number of the position, the input variable “row” represents the row number of the position and the input variable “value” represents the residual value. The input variable “fi_kind” is a three-digital integer. It stands for the kind of field, which decides the array in which the residual lies. The first digit represents the type of field element, which is the same as that in “ind_kind” above. The last two digits represent the type of equation, “51” stands for the static part of the *x*-momentum equation, “52” stands for the dynamic part of the *x*-momentum equation, “53” stands for the static part of the *y*-momentum equation, “54” stands for the dynamic part of the *y*-momentum equation, “55” stands for the static part of the *z*-momentum equation, “56” stands for the dynamic part of the *z*-momentum equation, “57” stands for the mass conservation equation and “58” stands for the concentration equation. Finally, “eq_i”, “eq_j”, and “eq_k” are the equation coordinates in the local equation field, which are used to locate the position of the residual in the array.

**Fig 14:**
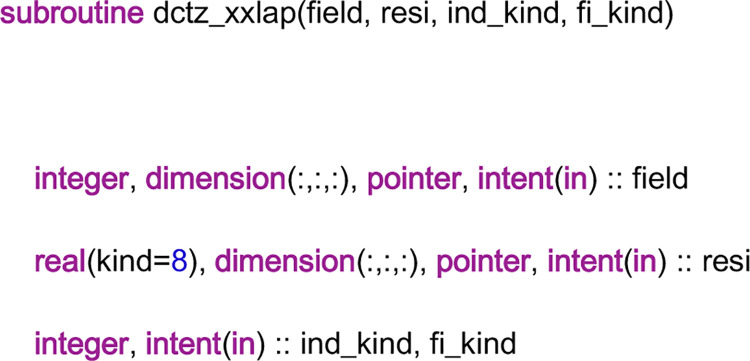
The head part of the code of the subroutine “dctz_xxlap”.

Now, we will study some details of the subroutine “dctz_xxlap”. When the subroutine is called, it is stipulated that the coefficients and the right-hand-side values of the AEs are already computed by the coefficient library. Thus, the discretization procedure is to put the coefficients and the right-hand-side values of the AEs into the corresponding arrays in the linear system library, for example, the arrays in Fig 5. The head part of this subroutine code can be given in Fig 14. From the figure, it can be seen that there are four input variables in the parameter list. The input variable “field” has the same meaning as “velx” in the subroutine “Resi_xmom_vx”, and the input variable “resi” has the same meaning as that in the subroutine “Resi_xmom_vx”. When an entry of the array “field” is set to one, the residuals of the AEs that contain the entry are sent to the coefficient storage arrays in the linear system library. However, to locate the positions of the residuals in the arrays, the information of the field element and the coefficient matrix should be known. Thus, the input variables “ind_kind” and “fi_kind” are needed, which have the same meaning as before. In particular, ind_kind = 111, and fi_kind = 151 or 152 in this condition.

### 6.6 The evaluation of Masor

Different from the former code-developing pattern in which the entire code should be written from the beginning, the general framework has already implemented most functions of the code, and therefore Masor can be implemented easily and quickly with the general framework. Masor has been applied in many cases of matrix acidization simulations. In the following discussion, those cases using Masor are retold for the purpose of evaluation. One evaluation is to verify the correctness and effectiveness of Masor, and the other evaluation is to show the good parallel performance of Masor. Since the success of Masor has been demonstrated in those works, the corresponding numerical experiments will not be repeated in this work. It is emphasized that in those works, Masor is just a tool to investigate the reasonability of the mathematical model and the effectiveness of the numerical algorithms, and Masor itself, and the general framework behind Masor, have never been the point of interest.

In Section 5.3 of [42], the authors used Masor to perform simulations and compared the numerical results of Masor with the work [45]. In that case, a matrix of 0.04 m × 0.04 m × 0.1 m, and a grid of 36 × 36 × 90 cells are used to discretize the matrix domain. An acid flow is injected into the matrix from the 0.04 m × 0.04 m face to produce an oil production channel, and the output is at the other 0.04 m × 0.04 m face. Except for the two faces, the other faces of the matrix are closed. The acid injection velocity is increased from 1.04 × 10^−7^ m/s to 1.04 × 10^−4^ m/s, and an acid-efficiency curve can be drawn from the simulation results. From the results, it is concluded that the two acid-efficiency curves of works [42, 45] match each other well. In addition, both works indicate that the optimal injection velocity is 1.04 × 10^−6^ m/s. Moreover, five different dissolution patterns in the work [45] can be reproduced by Masor in [42]. Thus, the correctness and effectiveness of Masor’s method are proven by [42]. It is acknowledged that the other works ([37–42, 46]) have also done many verification works, and we will not repeat them here.

In addition, Section 7 of [42] evaluates the parallel performance of Masor. There, the numerical experiment in the last paragraph is carried out with different numbers of processors, 9, 18, 36, 72 and 144. The percentage of solver time over run time is more than 98%, and therefore, it is the parallel performance of the solver that determines the parallel performance of Masor. In [42], the authors used the solver MUMPS. When the number of processors is increased from 9 to 36, the speedup is more or less linear. However, when the number of processors is increased further, a limited speedup can be achieved. This phenomenon can also be observed in other applications of MUMPS [47].

## 7. Conclusion

In this work, a field-based general framework to simulate fluids in parallel is suggested. Based on the experience from the fluid simulations, it is seen that all kinds of operations in the simulations can be attributed to the operations in the fields. With this philosophy, different fluid simulations can be deemed to follow the same kind of pattern, which necessitates building a general framework for all types of fluid simulations, i.e., the field-based general framework. As a result, most of the common operations in different simulations can be finished by the code of the framework, and the development of new simulators can be done easily and quickly.

The idea of a field-based general framework is summarized as follows—Due to the conservation laws, there are many common terms among the equations of fluid simulations, such as the divergence term and the Laplace term, so that the variables in the equations have common discretization patterns that are recognized by the discretization library. It is emphasized that the discretization patterns are established with the prerequisite that the equations and the variables are discretized on the field elements, i.e., the discretization patterns are field-based. After discretization, the PDEs become the AEs, and the variables become the DVs. Before assembling the AEs into a linear system, the coefficients of the AEs should be known. It is observed that the coefficient of a DV in an AE is in reality the residual of the AE, which is done by setting the DV as one and the other DVs in the AE as zero. This coefficient computing method is implemented by the coefficient library. Considering that all the AEs and DVs are imposed on the field elements, the coefficient library is also field-based. Then, the linear system library is applied to compute the indices of the residuals and are filled into the linear system. It is noted that the indices of the residuals are also the indices of the field elements, and therefore, the linear system library is field-based. There is no parallelization library to parallelize the field-based general framework. Instead, the parallel philosophy is integrated into the three libraries mentioned above. In parallelization, domain decomposition is performed to decompose the irregular domain into many regular subdomains, and then each subdomain is allocated to each processor. The size of the subdomains should be more or less the same to guarantee a load balance among the processors. With the domain decomposition strategy, the equation fields and the variable fields in the entire domain are decomposed into many local fields on the processors. Moreover, methods to convert the local index of the field element to its global index is another task in parallelization.

With the field-based general framework, the code of the decoupled scheme for the matrix acidization simulation, i.e., Masor, can be developed quickly. Matrix acidization is an operation in reservoirs, in which acid flow is injected into the matrix to produce an oil production channel. Thus, it is an application of computational fluid dynamics, and its code can be developed by the framework. From the simulation results, it is seen that Masor gives reasonable outputs, which is a demonstration of the correctness and effectiveness of the framework.

It is emphasized that the field-based general framework introduced in this work is based on regular grids, finite difference methods and linear systems. However, the philosophy of this framework can also be applied to irregular grids, finite element methods, finite volume methods, nonlinear systems, etc., and therefore the boundary of this framework can be further expanded. Additional new subroutines should be added into the libraries to cope with those conditions, which is work left for the future.

## Supporting information

S1 Code Makefile.mac. https://doi.org/10.6084/m9.figshare.16974196.v1.
(TXT)

S2 Code Infile.F90. https://doi.org/10.6084/m9.figshare.16974232.v1.
(TXT)

S3 Code Model.F90. https://doi.org/10.6084/m9.figshare.16974214.v1.
(TXT)

S4 Code Driver.F90. https://doi.org/10.6084/m9.figshare.16974250.v1.
(TXT)

S5 Code GlobalData.F90. https://doi.org/10.6084/m9.figshare.16974202.v1.
(TXT)

S6 Code ExportResults.F90. https://doi.org/10.6084/m9.figshare.16974226.v1.
(TXT)

S7 Code Resi.F90. https://doi.org/10.6084/m9.figshare.16974193.v1.
(TXT)

S8 Code ConstructMat.F90. https://doi.org/10.6084/m9.figshare.16974253.v1.
(TXT)

S9 Code Export2Matlab.F90. https://doi.org/10.6084/m9.figshare.16974199.v1.
(TXT)

S10 Code Export2tecplot.F90. https://doi.org/10.6084/m9.figshare.16974205.v1.
(TXT)

## References

[1] Kajishima Takeo, and Taira Kunihiko. "Computational fluid dynamics." Cham: Springer International Publishing 1 (2017).

[2] Tu Jiyuan, Guan Heng Yeoh, and Chaoqun Liu. Computational fluid dynamics: a practical approach. Butterworth-Heinemann, 2018.

[3] Demmel James. "LAPACK: A portable linear algebra library for high‐performance computers." Concurrency: Practice and Experience 3.6 (1991): 655–666.

[4] Davis Timothy A. "UMFPACK version 5.2. 0 user guide." University of Florida 25 (2007).

[5] Amestoy Patrick R., Duff Iain S., and L’excellent J-Y. "Multifrontal parallel distributed symmetric and unsymmetric solvers." Computer methods in applied mechanics and engineering 184.2–4 (2000): 501–520.

[6] Falgout, Robert D., and Ulrike Meier Yang. "hypre: A library of high performance preconditioners." International Conference on Computational Science. Springer, Berlin, Heidelberg, 2002.

[7] Abou-Kassem, Jamal Hussein, Syed Mohammad Farouq-Ali, and Rafiq Islam M. Petroleum Reservoir Simulations. Elsevier, 2013.

[8] Lie Knut-Andreas. An introduction to reservoir simulation using MATLAB/GNU Octave: User guide for the MATLAB Reservoir Simulation Toolbox (MRST). Cambridge University Press, 2019.

[9] Islam, Rafiqul M., Abou-Kassem J. H., and Farouq-Ali S. M. Petroleum Reservoir Simulation: The Engineering Approach. Elsevier, 2020.

[10] Ning Like, et al. "A review of fully coupled atmosphere-hydrology simulations." Journal of Geographical Sciences 29.3 (2019): 465–479.

[11] Dueben Peter D., et al. "Global simulations of the atmosphere at 1.45 km grid-spacing with the Integrated Forecasting System." Journal of the Meteorological Society of Japan. Ser. II (2020).

[12] Du Chuanbin, and Liang Dong. "An efficient S-DDM iterative approach for compressible contamination fluid flows in porous media." Journal of Computational Physics 229.12 (2010): 4501–4521.

[13] Ahadi Mina, Bergstrom Donald J., and Kerry Anne Mazurek. "Computational Fluid-Dynamics Modeling of the Flow and Sediment Transport in Stormwater Retention Ponds: A Review." Journal of Environmental Engineering 146.9 (2020): 03120008.

[14] Mian, Haris Hameed, Gang Wang, and Muhammad Aamir Raza. "Application and validation of HUNS3D flow solver for aerodynamic drag prediction cases." Proceedings of 2013 10th International Bhurban Conference on Applied Sciences & Technology (IBCAST). IEEE, 2013.

[15] Ma, Boping, et al. "Near field sonic boom analysis with HUNS3D solver." 55th AIAA Aerospace Sciences Meeting. 2017.

[16] Jin Haoqiang, Frumkin Michael, and Yan Jerry. "Automatic generation of OpenMP directives and its application to computational fluid dynamics codes." International Symposium on High Performance Computing. Springer, Berlin, Heidelberg, 2000.

[17] Thomas Jeffrey, et al. "Unsteady flow computation using a harmonic balance approach implemented about the OVERFLOW 2 flow solver." 19th AIAA Computational Fluid Dynamics. 2009. 4270.

[18] Derlaga Joseph M., Jackson Charles W., and Buning Pieter G. "Recent Progress in OVERFLOW Convergence Improvements." AIAA Scitech 2020 Forum. 2020.

[19] Rego F., et al. "FIRE PARADOX: an innovative approach of integrated wildland fire management–a joint European initiative." Proceeding of 4th International Wildland Fire Conference. 2007.

[20] Fernandes Paulo M., Rego Francisco C., and Rigolot Eric. "The FIRE PARADOX project: towards science-based fire management in Europe." Forest ecology and management 261.12 (2011): 2177–2178.

[21] Berger Marsha J., et al. "Performance of a new CFD flow solver using a hybrid programming paradigm." Journal of Parallel and Distributed Computing 65.4 (2005): 414–423.

[22] Jin Haoqiang, et al. "High performance computing using MPI and OpenMP on multi-core parallel systems." Parallel Computing 37.9 (2011): 562–575.

[23] Hawkes J., et al. "Performance analysis of massively-parallel computational fluid dynamics." (2014).

[24] Gourdain N., et al. "High performance parallel computing of flows in complex geometries: I. methods." Computational Science & Discovery 2.1 (2009): 015003.

[25] Gourdain N., et al. "High performance parallel computing of flows in complex geometries: II. Applications." Computational Science & Discovery 2.1 (2009): 015004.

[26] Yao Jixian, et al. "Unsteady flow investigations in an axial turbine using the massively parallel flow solver TFLO." 39th Aerospace Sciences Meeting and Exhibit. 2001.

[27] Jia Rongguang, and Bengt Sundén. "Parallelization of a multi-blocked CFD code via three strategies for fluid flow and heat transfer analysis." Computers & fluids 33. 1 (2004): 57–80.

[28] Chen Goong, et al. "OpenFOAM for computational fluid dynamics." Notices of the AMS 61.4 (2014): 354–363.

[29] Constant Eddy, et al. "An immersed boundary method in OpenFOAM: verification and validation." Computers & Fluids 157 (2017): 55–72.

[30] Basermann Achim, et al. "HICFD: highly efficient implementation of CFD codes for HPC Many-Core architectures." Competence in High Performance Computing 2010. Springer, Berlin, Heidelberg, 2011. 1–13.

[31] Shang Zhi. "High performance computing for flood simulation using Telemac based on hybrid MPI/OpenMp parallel programming." International Journal of Modeling, Simulation, and Scientific Computing 5.04 (2014): 1472001.

[32] Dong Suchuan, and George Em Karniadakis. "Dual-level parallelism for high-order CFD methods." Parallel Computing 30.1 (2004): 1–20.

[33] Vajdi Mohammad, et al. "A review on the Comsol Multiphysics studies of heat transfer in advanced ceramics." Journal of Composites and Compounds 2.2 (2020): 35–43.

[34] Zandi Soma, Saxena Prateek, and Gorji Nima E. "Numerical simulation of heat distribution in RGO-contacted perovskite solar cells using COMSOL." Solar Energy 197 (2020): 105–110.

[35] Ayguade Eduard, et al. "Employing nested OpenMP for the parallelization of multi-zone computational fluid dynamics applications." 18th International Parallel and Distributed Processing Symposium, 2004. Proceedings. IEEE, 2004.

[36] de Supinski Bronis R., et al. "The ongoing evolution of openmp." Proceedings of the IEEE 106.11 (2018): 2004–2019.

[37] Wu Yuanqing, Salama Amgad, and Sun Shuyu. "Parallel simulation of wormhole propagation with the Darcy–Brinkman–Forchheimer framework." Computers and Geotechnics 69 (2015): 564–577.

[38] Wu Yuanqing. Parallel Reservoir Simulations with Sparse Grid Techniques and Applications to Wormhole Propagation. Diss. 2015.

[39] Kou Jisheng, Sun Shuyu, and Wu Yuanqing. "Mixed finite element-based fully conservative methods for simulating wormhole propagation." Computer Methods in Applied Mechanics and Engineering 298 (2016): 279–302.

[40] Kou Jisheng, Sun Shuyu, and Wu Yuanqing. "A semi-analytic porosity evolution scheme for simulating wormhole propagation with the Darcy–Brinkman–Forchheimer model." Journal of Computational and Applied Mathematics 348 (2019): 401–420.

[41] Wu Yuanqing, and Ye Maoqing. "A Newton’s second law abided Darcy-Brinkman-Forchheimer framework in matrix acidization simulation." International Conference on Computational & Experimental Engineering and Sciences. Springer, Cham, 2019.

[42] Wu Yuanqing, et al. "Thermodynamically consistent Darcy–Brinkman–Forchheimer framework in matrix acidization." Oil & Gas Science and Technology–Revue d’IFP Energies nouvelles 76 (2021): 8.

[43] Pacheco Peter. Parallel programming with MPI. Morgan Kaufmann, 1997.

[44] Gropp William, et al. Using MPI: portable parallel programming with the message-passing interface. Vol. 1. MIT press, 1999.

[45] Akanni Olatokunbo O., Nasr-El-Din Hisham A., and Deepak Gusain. "A computational Navier-Stokes fluid-dynamics-simulation study of wormhole propagation in carbonate-matrix acidizing and analysis of factors influencing the dissolution process." SPE Journal 22.06 (2017): 2049–2066.

[46] Wu Yuanqing, et al. "A Decoupled Scheme to Solve the Mass and Momentum Conservation Equations of the Improved Darcy-Brinkman-Forchheimer Framework in Matrix Acidization." arXiv preprint arXiv:2008.03268 (2020).

[47] Raju Mandhapati P. "Parallel computation of finite element Navier-Stokes codes using MUMPS solver." arXiv preprint arXiv:0910.1845 (2009).

